# Evidence of hybridization, mitochondrial introgression and biparental inheritance of the kDNA minicircles in *Trypanosoma cruzi I*

**DOI:** 10.1371/journal.pntd.0007770

**Published:** 2020-01-31

**Authors:** Fanny Rusman, Noelia Floridia-Yapur, Paula G. Ragone, Patricio Diosque, Nicolás Tomasini

**Affiliations:** Unidad de Epidemiología Molecular (UEM), Instituto de Patología Experimental, Universidad Nacional de Salta-CONICET, Salta, Salta, Argentina; Universiteit Antwerpen, BELGIUM

## Abstract

**Background:**

Genetic exchange in *Trypanosoma cruzi* is controversial not only in relation to its frequency, but also to its mechanism. Parasexual genetic exchange has been proposed based on laboratory hybrids, but population genomics strongly suggests meiosis in *T*. *cruzi*. In addition, mitochondrial introgression has been reported several times in natural isolates although its mechanism is not fully understood yet. Moreover, hybrid *T*. *cruzi* DTUs (TcV and TcVI) have inherited at least part of the kinetoplastic DNA (kDNA = mitochondrial DNA) from both parents.

**Methodology/Principal findings:**

In order to address such topics, we sequenced and analyzed fourteen nuclear DNA fragments and three kDNA maxicircle genes in three TcI stocks which are natural clones potentially involved in events of genetic exchange. We also deep-sequenced (a total of 6,146,686 paired-end reads) the minicircle hypervariable region (mHVR) of the kDNA in such three strains. In addition, we analyzed the DNA content by flow cytometry to address cell ploidy. We observed that most polymorphic sites in nuclear loci showed a hybrid pattern in one cloned strain and the other two cloned strains were compatible as parental strains (or nearly related to the true parents). The three clones had almost the same ploidy and the DNA content was similar to the reference strain Sylvio (a nearly diploid strain). Despite maxicircle genes evolve faster than nuclear housekeeping ones, we detected no polymorphisms in the sequence of three maxicircle genes showing mito-nuclear discordance. Lastly, the hybrid stock shared 66% of its mHVR clusters with one putative parent and 47% with the other one; in contrast, the putative parental stocks shared less than 30% of the mHVR clusters between them.

**Conclusions/significance:**

The results suggest a reductive division, a natural hybridization, biparental inheritance of the minicircles in the hybrid and maxicircle introgression. The models including such phenomena and explaining the relationships between these three clones are discussed.

## Author summary

Chagas disease, an important public health problem in Latin America, is caused by the parasite *Trypanosoma cruzi*. Despite being a widely studied parasite, several questions on the biology of genetic exchange remain unanswered. Population genomic studies have inferred meiosis in *T*. *cruzi*, but this cellular division mechanism has not been observed in laboratory yet. In addition, previous results suggest that mitochondrial DNA (called kDNA) may be inherited from both parents in hybrids. Here, we analyzed a hybrid strain and its potential parents to address the mechanisms of genetic exchange at nuclear and mitochondrial levels. We observed that the hybrid strain had heterozygous patterns and DNA content compatible with a meiosis event. Also, we observed that the evolutionary histories of nuclear DNA and kDNA maxicircles were discordant and that the three strains shared identical DNA sequences. Mitochondrial introgression of maxicircle DNA from one genotype to another may explain this observation. In addition, we demonstrated that the hybrid strain shared kDNA minicircles with both parental strains. Our results suggest that hybridization implied meiosis and biparental inheritance of the kDNA. Further research is required to address such phenomena in detail.

## Introduction

The trypanosomatids cause devastating diseases around the world [1–3]. *Trypanosoma cruzi*, *Trypanosoma brucei* and *Leishmania spp*. species are the main human pathogens among the trypanosomatids, although other members of this family are also relevant [4, 5]. Genetic exchange has been proposed naturally occurring in these species [6], although its frequency is still debated [7–16]. Genetic exchange by meiosis has been described in *T*. *brucei* [17, 18] and *Leishmania* [19]. In spite of being recently proposed by genomic studies [14, 20] meiosis has not been reported for *T*. *cruzi* in the laboratory yet. Alternatively, experimental tetraploid or sub-tetraploid hybrids were observed in laboratory conditions [21, 22]. The fusion of diploid cells to form a tetraploid and posterior random loss of chromosomes slowly returning to diploidy was proposed as a model to explain genetic exchange in this species [21]. This mechanism is similar to parasexuality in *Candida albicans* [23]. Interestingly, an homozygous state in one-third of the chromosomes in parasexual hybrids is expected due to random loss of both chromosomes of the same parent [24]. In *T*. *cruzi*, with more than 40 pairs of chromosomes [25], the presence of chromosomes in a homozygous state in hybrid strains would be evident if parasexuality was indeed the mechanism of genetic exchange. There are six major *T*. *cruzi* lineages (named TcI to TcVI) [26, 27]. Particularly, the natural hybrids TcV and TcVI appear to be heterozygous in most loci which is unexpected under the parasexual model [28, 29] but fits the hypothesis of a reductive division prior to cell fusion. A recent study based on sequencing of 45 TcI genomes from a restricted geographical area showed that both mechanisms (sexual and parasexual) may occur in natural populations [20].

The inheritance of the kDNA in hybrid strains also remains as an open question on the reproductive biology of *T*. *cruzi*. Uniparental inheritance of maxicircles in natural occurring hybrids [30] and in laboratory-made hybrids was previously suggested [21]. The hybrid DTUs TcV and TcVI share nuclear genomes with the parental DTUs TcII and TcIII; whereas maxicircle sequences in both hybrid DTUs are derived from TcIII only [31]. On the other hand, technical limitations caused by the great sequence variability of minicircle has hindered the study of inheritance of these molecules [32]. Recently, we studied the hypervariable region of the minicircles (mHVR) by deep sequencing and observed that TcV and TcVI share minicircles with both parental DTUs (TcII as TcIII) and not just with TcIII, as observed in maxicircles. Based on these results we proposed that minicircle inheritance may be biparental in natural occurring hybrids [33]. Biparental inheritance of the kDNA has also been proposed for *T*. *brucei* [34–36].

Another question related to the mechanisms of genetic exchange in *T*. *cruzi* is the mito-nuclear discordance observed in some DTUs. This phenomenon can be described as a significant difference in levels of differentiation between nuclear and mitochondrial markers, where nuclear phylogeny is more structured (higher number of and longer branches) than mitochondrial phylogeny (kDNA phylogeny in this case) [37]. In *T*. *cruzi*, mito-nuclear discordance is clearly observed in TcIII. According to nuclear loci, TcIII is nearly related to TcI; although maxicircle sequences cluster TcIII with TcIV from South America [31, 38]. In addition, mito-nuclear discordance was also observed within TcI [39–41]. This phenomena in *T*. *cruzi* could be caused by mitochondrial introgression i.e. successive backcrosses of a hybrid with one of the parents.

In previous studies, we described a TcI strain (TEDa2cl4) isolated from Chaco Province, Argentina, as a potential hybrid. TEDa2cl4 presented some heterozygous patterns in isoenzymatic loci [42], a high number of heterozygous SNPs in the spliced-leader intergenic region [43] and heterozygous patterns in nuclear loci revealed by MLST [29, 44]. We isolated—within the same restricted geographical area—two strains (TEV55cl1 and PalDa20cl3) that could be potential parents of such hybrid, considering also isoenzymatic loci [42], the SL-IR [43] and MLST [29, 44]. Considering the above, this strain triplet is an interesting opportunity to address the mechanism of genetic exchange in *T*. *cruzi*. Here, we analyzed the DNA sequence for 14 nuclear loci of such strains along with 3 maxicircle regions and around 6.1 million paired-end reads of the mHVRs. We also addressed ploidy of the strains by flow cytometry.

## Materials and methods

### Strains

Three laboratory cloned stocks isolated from Chaco province, Argentine, and belonging to TcI, were analyzed. The stocks characteristics are summarized in Table 1. In addition, the TcI strains Sylvio and TEV91cl5 were also cultured and used as controls. The stocks were maintained in liver infusion-tryptose (LIT) medium supplemented with 10% fetal bovine serum. Parasites in exponential growth phase were harvested by centrifugation (800 xg, 10 min, 4 °C) for DNA extraction and flow cytometry. DNA was extracted by using a commercial kit (Inbio Highway).

**Table 1:** TcI stocks used in this study to address hybridization and kDNA inheritance.

Stock	Host	Geographical origin (Chaco Province, Argentine)	SL-IR group^1^ / MLST cluster^2^
PalDa20cl3	*Didelphis albiventris*	EL PALMAR, 12 de Octubre	Chaco-3 / Chaco-3
TEDa2cl4	*Didelphis albiventris*	TRES ESTACAS, Chacabuco	Chaco-3 / potential hybrid^3^
TEV55cl1	*Triatoma infestans*	TRES ESTACAS, Chacabuco	Chaco-2 /Chaco-2

^1^. Group according to Spliced Leader Intergenic Region (SL-IR) sequence [43].

^2^. Cluster according sequence of 8 nuclear gene fragments as analyzed in [44].

^3^. MLST analysis suggested TEDa2cl4 is a hybrid between Chaco-2 and Chaco-3 Clusters [44].

### Sequencing of nuclear and maxicircle gene fragments

The primers used to amplify eight nuclear regions and three maxicircle gene fragments are shown in Table 2. Primer sequences described for first time in this paper were estimated by using Primer3Plus [45] with default parameters. PCRs were carried out in reaction volumes of 50 μl containing 50 ng of DNA; 0.2 μM of each primer, 1 U of goTaq DNA polymerase (Promega), 10 μl of 5X buffer (supplied with the goTaq polymerase) and a 50 μM concentration of each dNTP (Promega). Cycling conditions were as follow: 3 min at 94 °C followed by 30 cycles of 94 °C for 30 seconds; 50 °C for 30 seconds; and, 72 °C for 30 seconds, with a final extension at 72 °C for 10 minutes. Amplified fragments were precipitated with 70% ethanol and sequenced on both strands in an ABI PRISM_310 Genetic Analyzer (Applied Biosystems). In addition, sequences from genes leucine aminopeptidase (*LAP*), glucose-6-phosphate isomerase (*GPI*), glutathione peroxidase (*GPX*), pyruvate dehydrogenase E1 component alpha subunit (PDH), 3-hydroxy-3-methylglutaryl-CoA reductase (HMCOAR) and small GTP-binding protein rab7 (GTP) were obtained in a previous work [44].

**Table 2:** Gene fragments, genomic location and primers used in this study.

Gene	Genomic location on CLBrener	Forward primer	Reverse primer	Reference
Hypothetical protein	TcChr6-S: 151495–152340	GTTCCACTTCCTCCATTTGC	GCATGGCTCTGAAACAACAA	This paper
Hypothetical protein	TcChr6-S: 152483–153291	TTCCATTTGCTCCTCTCGTT	TGTCTCCCTTTGGTGGTTACA	This paper
glucose-6-phosphate isomerase	TcChr6-S:153784–154584	AGGCCATTTGTTGAAGAATCA	GTCCGCCATTGGTTTATCTG	This paper
glucose-6-phosphate isomerase	TcChr6-S:154577–154981	CGCCATGTTGTGAATATTGG	GGCGGACCACAATGAGTATC	[29, 44]
glucose-6-phosphate isomerase	TcChr6-S:155006–155562	TCCATTCACCTCTCCTGACC	CCTGAAGGATTTGACGGTTC	This paper
ATP synthase subunit	TcChr6-S:155935–156336	AAGCTCAAATAATCCCTCAACG	AAAGATATAATGCCCACAATCC	This paper
Hypothetical protein	TcChr6-S:156512–156877	AAGCAGGCAACTTTGAATCC	TCAACAAGGACGGGAGAGC	This paper
Small GTP-binding protein Rab7 (GTP)	TcChr12-S: 436418–436978	TGTGACGGGACATTTTACGA	CCCCTCGATCTCACGATTTA	[29, 44]
Leucine Aminopeptidase (LAP)	TcChr27-P: 223436–223825	TGTACATTGTGCTTGGCTGAG	GCTGAGGTGATTAGCGACAAA	[29, 44]
3-Hidroxi-3-metilglutaril-CoA reductase (*HMCOAR*)	TcChr32-S: 852060–852613	AGGAGGCTTTTGAGTCCACA	TCCAACAACACCAACCTCAA	[29, 44]
glutathione peroxidase (*GPX*)	TcChr35-S: 417886–418172	TGCAAAAGGGACATGTGATG	CCCGGTGTAATTGGAGAGTG	This paper
glutathione peroxidase (*GPX*)	TcChr35-S: 418148–418507	CGTGGCACTCTCCAATTACA	AATTTAACCAGCGGGATGC	[29, 44]
pyruvate dehydrogenase E1 component alpha subunit (*PDH*)	TcChr40-S: 1048833–1049323	GGGGCAAGTGTTTGAAGCTA	AGAGCTCGCTTCGAGGTGTA	[29, 44]
Hypothetical protein	TcChr40-S: 1050394–1051576	TGGGTTTCTTGGATTTGGAC	GGCGTCTCTTACACCGACAC	This paper
NADH dehydrogenase subunit 1 (ND1)	Maxicircle	GCACTTTCTGAGATAATCGAAAA	TTAATCTTATCAGGRTTTGTTAGCC	This paper^1^
Cytochrome c Oxidase II (COII)	Maxicircle	GTTATTATCTTTTGTTTGTTTTGTGTG	AACAATTGGCATGAATCCATGT	This paper^1^
Mitochondrial unidentified Reading frame 1 (MURF-1)	Maxicircle	ACACCCATCCATTCTTCA	CMTTTGATCTATTGTGAYTAACGGT	This paper^1^

^1^. DNA fragment sequenced in this study, but primers designed by [39].

### Flow cytometry

Pellets containing 1x10^7^ parasites were resuspended in 300 μl of PBS and 700 μl of ice-cold methanol. The tube was gently inverted and incubated on ice for 10 min. Fixed parasites were centrifuged at 800 xg and washed in 5 ml of PBS two times. The pellet was resuspended in 1 ml of PBS. Propidium Iodide and RNAse A were added to a final concentration of 30 μl/ml and 10 μg/ml, respectively, and incubated 45 min at 37 °C. Samples were analyzed in a FACS Canto II in FL2 channel. Around 50,000 events were recorded for each sample and the samples were analyzed by duplicate. Flowing software (http://flowingsoftware.btk.fi) was used for data analysis. After gating out debris and cell clumps the data were plotted as fluorescence area histograms. Gates were created for G1/0 (2*n*) peaks and for G2/M (4*n*) peaks. Mean G1/0 values were used to infer relative DNA content. The coefficient of variation (CV) was recorded for each fluorescence peak. The DNA content for each stock was expressed relative to Sylvio strain, a nearly diploid strain [46].

### mHVR sequencing

The mHVRs for TEDa2cl4 were amplified according to the protocol and primers proposed in [33]. Amplicons were then purified using the magnetic beads Agencourt AMPure XP-PCR Purification (Beckman Genomics, USA). The concentration of the purified amplicons was controlled using Qubit Fluorometer 2.0 (Invitrogen, USA). The library was validated using the Fragment Analyzer system (Advanced Analytical Technologies, USA). The library was sequenced on an Illumina MiSeq using a 500 cycle v2 kit (Illumina, San Diego, USA). The mHVR reads from TEV55cl1 and PalDa20cl3 were previously published in [33].

### Bioinformatics

The reads were demultiplexed, trimmed and filtered according to [33]. After filtering, the sequences were clustered with the “pick_de_novo_otus.py” script in QIIME V1.9.1 [47]. The *de novo* approach groups sequences based on sequence identity using the *uclust* algorithm [48]. Default parameters were used, and sequences were clustered according to two identity thresholds—85% and 99%—in order to determine different mHVR clusters. Output tables were filtered at 0.005% using the “filter_otus_from_otu_table.py” script, in order to discard mHVR clusters with low abundance which are probably PCR or sequencing artifacts [49]. The remaining parameters of the “filter_otus_table.py” script were used by default. For each strain, the presence of a mHVR cluster was discarded when its abundance was lower than 20 read sequences. In order to compare stocks with different number of reads, the datasets were rarefacted to the number of reads in the smallest dataset (TEDa2cl4 in this case) by using the “multiple_rarefactions.py” script available in QIIME. This script creates a series of subsampled mHVR clusters by random sampling (without replacement) of the input mHVR clusters. The “jackknifed_beta_diversity.py” script was used to estimate compositional dissimilarity among stocks. Default parameters and the Bray-Curtis measure were chosen. The script calculates the beta diversity between each pair of previously resampled input strains, building a distance matrix. The distance matrix was visualized by using Principal Coordinate Analysis (PCoA).

### Phylogenetic analysis

In order to address mito-nuclear discordance, the uncorrected *p*-distance and a model corrected distance were calculated between pairs of strains (TEV55cl1-CL Brener, TEV55c1-Esmeraldo, CL Brener-Esmeraldo, TEV55cl1-TEV91cl5 and TEV55cl1-PalDa20cl3) for each gene fragment using MEGA v7 [50]. The CL Brener sequences for each nuclear gene fragment were obtained from CL Brener (non Esmeraldo) genome [25] from TriTryp database. The average distance (model corrected and uncorrected) and the standard deviation were calculated for nuclear and maxicircle DNA fragments in order to compare substitution rates. Model corrected distances were calculated using MEGA v7 software by selecting the best substitution model for each gene fragment and calculating the pairwise distance between strains according to the selected model. In addition, sequences for the 14 nuclear gene fragments and three maxicircle genes from TcI strains JRcl4 and Dm28c (available genomes in TriTryp) were included into the analyses in order to address mito-nuclear discordance in TcI. Nuclear and maxicircle sequences were concatenated in two independent alignments using MLSTest [51]. The concatenated sequences were analyzed using MEGA v7 to select the best substitution model and to build phylogenetic trees with 1,000 bootstrap replications.

### Accession numbers

Nuclear DNA sequences from [44] were downloaded from GenBank (KF264037, KF268604, KF268636, KF268620, KF264005, KF264021, JN129573, JN129606, JN129639, JN129507, JN129540, JN129672, JN129608, JN129509, JN129674, JN129641, JN129575, JN129542). Nuclear and maxicircle DNA sequences obtained in this paper are available in GenBank with the following accession numbers: MN413539-MN413581. The mHVR reads for the three strains are available the NCBI SRA database (BioProject ID: PRJNA514922).

## Results

### Analysis of fourteen nuclear loci and DNA content reveals that TEDa2cl4 is an almost diploid hybrid clone

**Fig 1:**
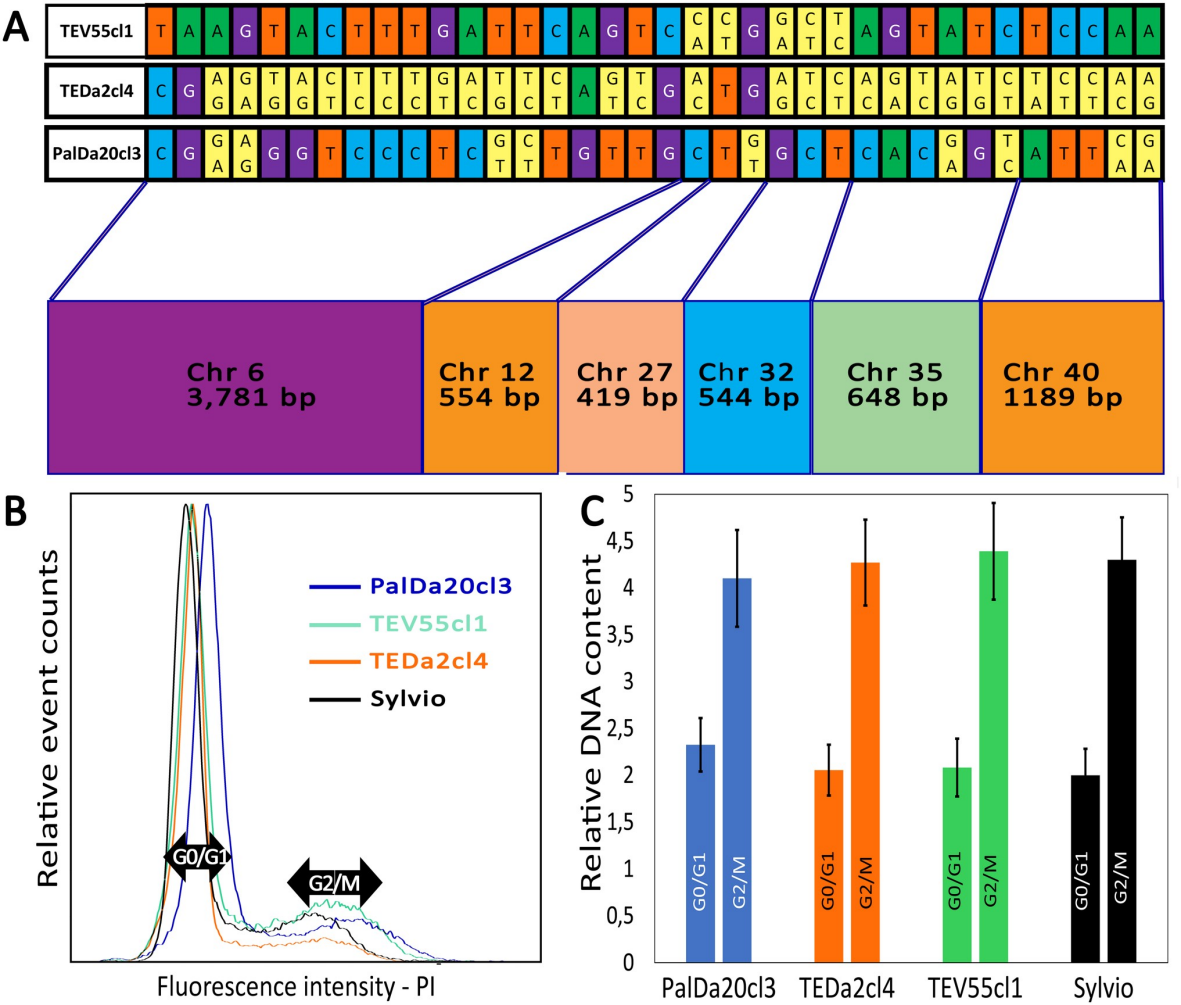
Polymorphic sites in 14 nuclear DNA fragments and flow cytometry analysis show TEDa2cl4 is a near-diploid hybrid strain. (A) polymorphic sites in 14 nuclear DNA fragments for the strains TEV55cl1, TEDa2cl4 and PalDa20cl3 showing heterozygous sites (yellow bases). (B) Proportion of cells with different DNA content measured by fluorescence intensity of propidium iodide in flow cytometry. (C) DNA content in different cell-cycle stages in relation to the nearly diploid strain Sylvio.

A total of 7,135 bases corresponding to 14 gene fragments on 6 chromosomes were analyzed in the stocks TEDa2cl4, PalDa20cl3 and TEV55cl1. The polymorphic sites for each fragment are shown on Fig 1A. The number of polymorphisms was low (36–0.5% of the total analyzed sites). Thirty (83.3%) polymorphic sites were heterozygous in TEDa2cl4. Moreover, in 29/30 (96.7%) of these heterozygous sites, the pattern was compatible with the hypothesis of PalDa20cl3 and TEV55cl1 as parents (or at least closely related to the parents). This result reinforces the hypothesis of TEDa2cl4 as a hybrid. Additionally, heterozygous SNPs in TEDa2cl4 were found in different chromosomes (Fig 1A). On the other hand, the percentage of heterozygous polymorphic sites was relatively lower in PalDa20cl3 (25%) and TEV55cl1 (13.9%) than in TEDa2cl4. The flow cytometry analysis for the three strains showed that they had similar DNA contents (Fig 1B) suggesting a reductive division as meiosis. Particularly, the DNA content of TEDa2cl4 (hybrid) and TEV55cl1 (putative parental) were almost identical. The estimated ploidy level is similar to the one obtained in the almost diploid reference strain Sylvio (Fig 1C). In addition, PalDa20cl3 showed the highest relative DNA content (2.3n expressed as the relative DNA content in relation to a half of the DNA content in Sylvio).

### Maxicircle genes analysis showed mito-nuclear discordance

**Fig 2:**
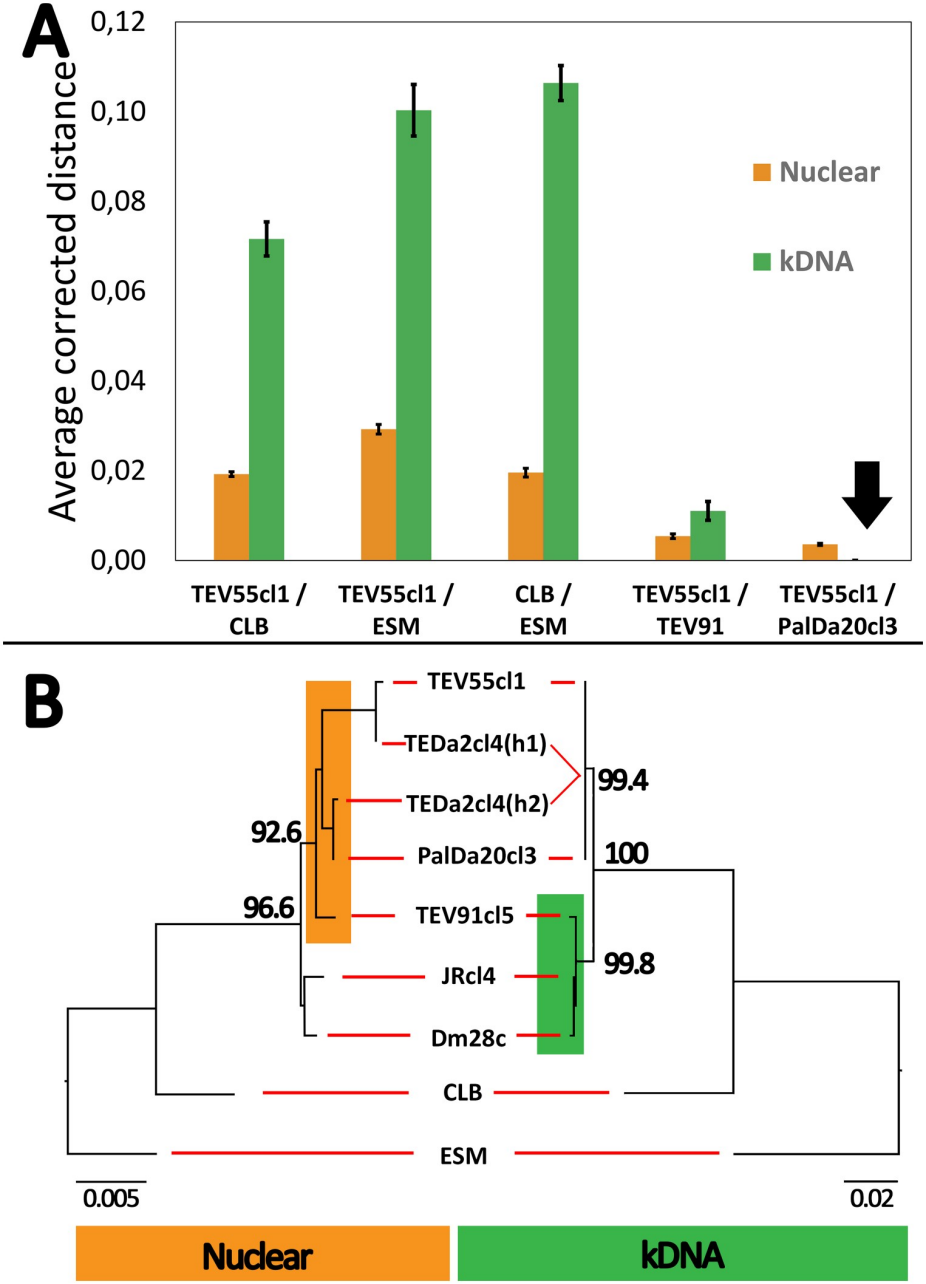
Phylogenetic analysis of nuclear and kDNA gene fragments reveals mito-nuclear discordance in TcI strains. (A) Comparison of averaged model-corrected distances among nuclear loci and kDNA loci. Sequences from TcI (TEV91cl5, TEV55cl1, PalDa20cl3), TcII (Esmeraldo-ESM), and TcIII (CLB, inferred from CL-Brener Non Esmeraldo genome) where used in order to address relative substitution rates. Most comparisons showed higher genetic distances for kDNA markers than for nuclear loci except for the comparison between TEV55cl1 and PalDa20cl3. Error bars represent the standard error of the mean corrected distance. (B) Nuclear phylogeny (left) and kDNA phylogeny (right) showing discordances between both markers. TEDa2cl4 haplotypes (h1 and h2) were manually inferred according the parental genotypes. The trees were estimated based on the best substitution model for concatenated genes. Values above branches indicate bootstrap support for 1,000 replicates. Note that location in different clusters of TEV91cl5 (highlighted) and different branch scales for both markers.

In contrast to the polymorphic sites observed in the nuclear loci, sequences of three maxicircle genes (1,335 bp) revealed no polymorphic sites among the three strains. This was an unexpected result because coding maxicircle genes mutate faster than nuclear coding genes. This is revealed in Fig 2A showing such different substitution rates among different DTUs and within TcI. When uncorrected *p*-distance or model corrected distances were considered for pairs of strains, a higher average substitution rate in maxicircle loci than in nuclear loci was observed for most comparisons (Fig 2A and S1 Fig). The exception was the pair TEV55cl1-PalDa20cl3, which had more substitutions at nuclear loci than at mitochondrial loci. This result indicates mito-nuclear discordance. A comparison between nuclear and mitochondrial phylogenetic trees also reveals the mito-nuclear discordance (Fig 2B). In addition, the trees also showed discordance considering other TcI strains suggesting that it is not an uncommon phenomenon.

### mHVR deep sequencing reveals that minicircles are biparentally inherited in TEDa2cl4

We performed deep sequencing of the mHVR amplicons for the three strains. The number of reads obtained for each strain is observed in Table 3. Sequences were clustered according to the percentage of sequence identity. Despite having no variation in the three maxicircle loci analyzed (Fig 2), the number of mHVR clusters in these strains was highly variable (Table 3). In addition, a rarefaction analysis showed that the number of clusters did not depend on the number of reads for each strain.

**Table 3:** Deep sequencing of the minicircle hypervariable region (mHVR) in three TcI strains.

	TEV55cl1	TEDa2cl4	PalDa20cl3
Number of paired reads	2,305,460	1,113,033	2,728,193
Filtered and merged	1,202,355	950,142	2,356,494
Number of mHVR clusters	285	217	350
Number of mHVR clusters rarefacted to 900,000 reads (standard deviation for 100 replicates)	284.8(0.4)	215.6(0.9)	346.8(0.4)

**Fig 3:**
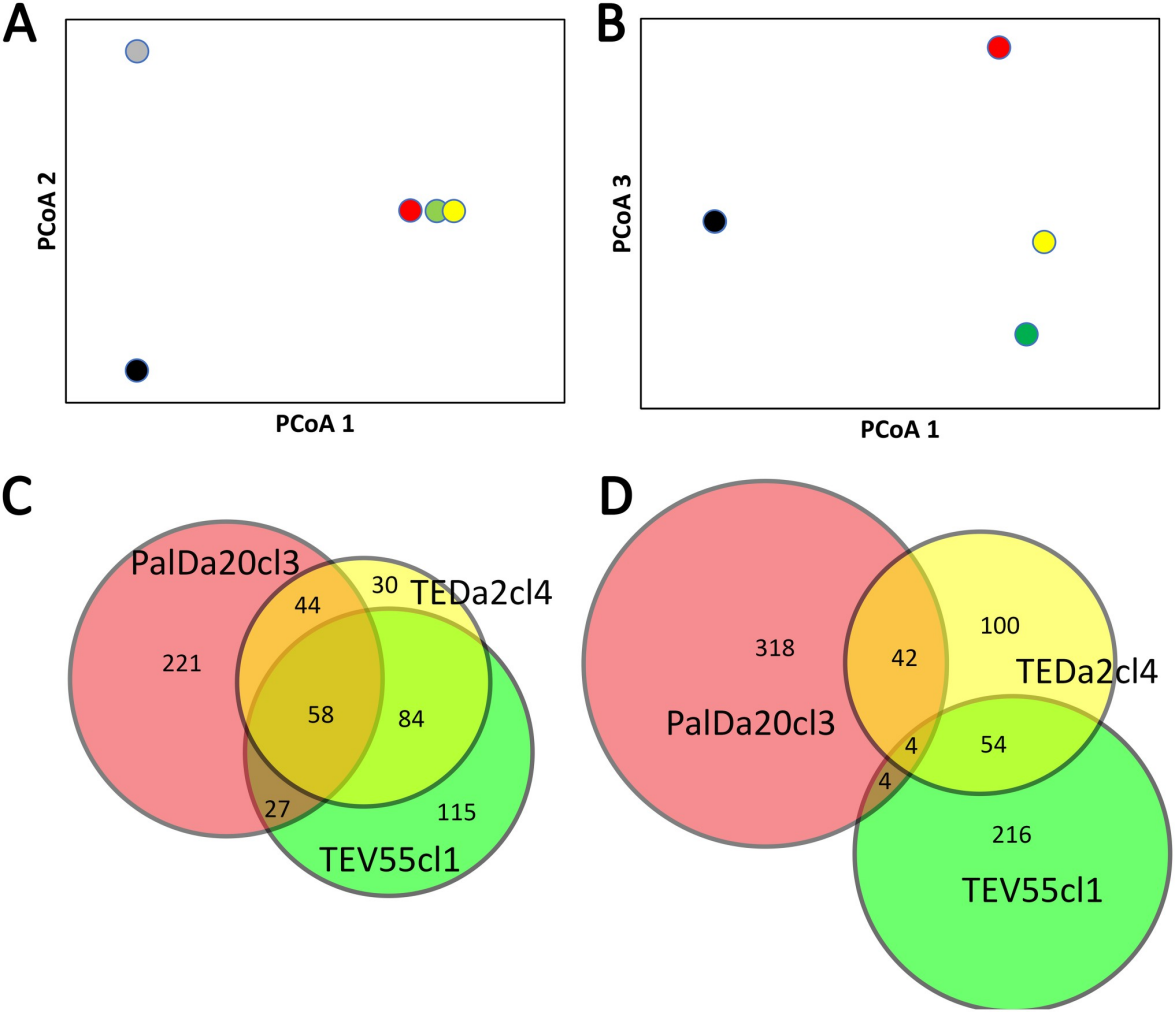
Amplicon sequencing of the mHVR reveals that minicircles of the hybrid strain TEDa2cl4 were biparentally inherited. (A-B) Principal Coordinates Analysis based on Bray-Curtis dissimilarities for clusters at 85% identity threshold showing the first three axes for PalDa20cl3 (red dot), TEV55cl1 (green dot), TEDa2cl4 (yellow dot) and the outgroup strains CANIIIcl1 (TcIV, grey dot) and X109/2 (TcIII, black dot). The three first axes explain 84% of the observed dissimilarities. (A) Axis 1 vs Axis 2, the first two axes show the separation among different DTUs. (B) the third axis shows the separation between TcI strains. Note that TEDa2cl4 was located between PalDa20cl3 and TEV55cl1. (C-D) Analysis of shared mHVR clusters between the three strains using Venn diagrams. Each circle represents the total number of mHVR clusters in a strain. Overlapped regions among circles represent shared mHVR clusters. (C) mHVR clusters defined at 85% of identity threshold showing few singletons in TEDa2cl4. (D) mHVR clusters defined at 99% identity threshold.

A Principal Coordinates Analysis (PCoA) (Fig 3A and 3B) of Bray-Curtis dissimilarities among strains revealed that TEDa2cl4 did not cluster closely to one of the parental-like strains as would be expected under the hypothesis of uniparental inheritance of minicircles. Conversely, PCoA showed that TEDa2cl4 located almost at the middle between both strains (Fig 3B). In addition, we analyzed shared clusters at different identity thresholds (Fig 3C and 3D). At 85% identity threshold, most of the mHVR clusters in TEDa2cl4 (84%) were observed in TEV55cl1 and/or PalDa20cl3 (Fig 3C) suggesting biparental inheritance of the minicircles in this hybrid. On the other hand, TEV55cl1 and PalDa20cl3 had higher percentages (40.4% and 63.1% respectively) of mHVR clusters which are singletons (i.e. clusters not shared with any other strain) (Fig 3C). We also analyzed shared mHVR clusters at a more restrictive clustering threshold (at 99% identity threshold). In this case, the number of mHVR clusters in TEDa2cl4 that were observed in PalDa20cl3 and/or TEV55cl1 was reduced to 50% (Fig 3D). This genetic divergence suggests that it is more likely that TEV55cl1 and PalDa20cl3 are not related to TEDa2cl4 in a recent time (i.e. they are not direct parents). At this threshold, TEV55cl1 and PalDa20cl3 shared very few mHVR clusters (8 clusters corresponding to 2.9% and 2.2%, respectively), although they still shared a significant percentage of mHVR clusters with the hybrid TEDa2cl4 (Fig 3D). Interestingly, from the mHVR clusters that TEDa2cl4 shared with any other strain, 54% were observed in TEV55cl1 but not in PalDa20cl3 and 42% were observed PalDa20cl3 but not in TEV55cl1. Such results strongly support that minicircle inheritance is biparental in the hybrid strain.

## Discussion

Genetic exchange has been previously reported in natural populations of *T*. *cruzi* [14, 20, 39, 40, 52–54]. In addition, genomic population studies proposed meiosis in this parasite [14, 20], although the experimental observation of gametes and laboratory meiotic hybrids is still elusive. In addition, the frequency of genetic exchange and how the population structure is maintained is still under debate [7–16, 20]. Here, we analyzed three TcI strains that previously showed evidence of genetic exchange events [29, 42, 43, 55]. In these three strains we analyzed DNA sequences at nuclear, maxicircle and minicircle levels. Interestingly, the results showed that the strain TEDa2cl4 would be the result of a hybridization between different TcI genotypes maintaining diploidy, which suggests a reductive division. The strains TEV55cl1 and PalDa20cl3 are, at least, genetically related to the parental genotypes of TEDa2cl4. In addition, analysis of maxicircle genes revealed mito-nuclear discordance and suggests mitochondrial introgression by backcrosses in TEV55cl1 and/or PalDa20cl3. Finally, we observed that the hybrid TEDa2cl4 shared minicircles (mHVRs) with both putative parental genotypes, which strongly suggests biparental inheritance of minicircles in hybrids.

The analysis of nuclear DNA fragments of at least five different chromosomes, revealed that TEDa2cl4 is highly heterozygous suggesting that it was maintained by clonal propagation. We have also detected other strains with the same genotype than TEDa2cl4 in the same area [55]. A recent paper revealed the existence of nuclear genetic exchange in natural populations of TcI in Arequipa, Peru [14]. The authors proposed by sequencing 123 TcI genomes that several rounds of inbreeding occurred after the origin of a highly heterozygous founder population. Our data revealed no evidence of inbreeding in TEDa2cl4 and the related strains. Moreover, our previous results for TcI in a restricted geographical area revealed well defined clusters with high linkage disequilibrium, congruence among different genetic markers and no geographical association [55]. However, another population genomic study in TcI stocks from Ecuador also showed that almost panmictic populations and clonal populations may coexist [20]. It is probable that the sexual exchange has been blocked in the hybrid TEDa2cl4 and the strain was maintained by clonal propagation, as observed in TcV and TcVI. The reduced number of mHVR clusters compared with its parents supports the above hypothesis since clonal propagation is expected to reduce minicircle diversity [56]. Such reproduction blockage may be related to hybrids that arose from genetically distant genotypes. However, mitochondrial introgression (caused by backcrosses) was observed between different DTUs [39–41] suggesting that hybrids from genetically distant genotypes may still reproduce sexually.

The mechanism of genetic exchange is still debated. The model of parasexuality proposes diploid fusion and posterior chromosome loss to return diploidy [21]. Fusion of diploid cells has been observed in the laboratory [21]. However, the returning from tetraploidy to diploidy was never observed in such hybrids [57]. Parasexuality appears to be infrequent in nature, although it could not be discarded from a population genomic study where several aneuploid stock were observed [20]. Despite this hybrid DTUs such as TcV and TcVI showed heterozygous patterns more compatible with meiosis and gamete fusion than with the model of parasexuality [24, 38, 57]. An expected result of the parasexual model is the existence of strains with high degrees of aneuploidy. Contrary to this, our results suggest meiosis since the hybrid TEDa2cl4 had a DNA content compatible with diploidy: TEDa2cl4 had the same DNA content than an almost diploid strain (Sylvio has 1/41 pairs of chromosomes in aneuploidy) [46]. However, sexual and parasexual modes of reproduction may both be occurring simultaneously in natural populations [20].

**Fig 4:**
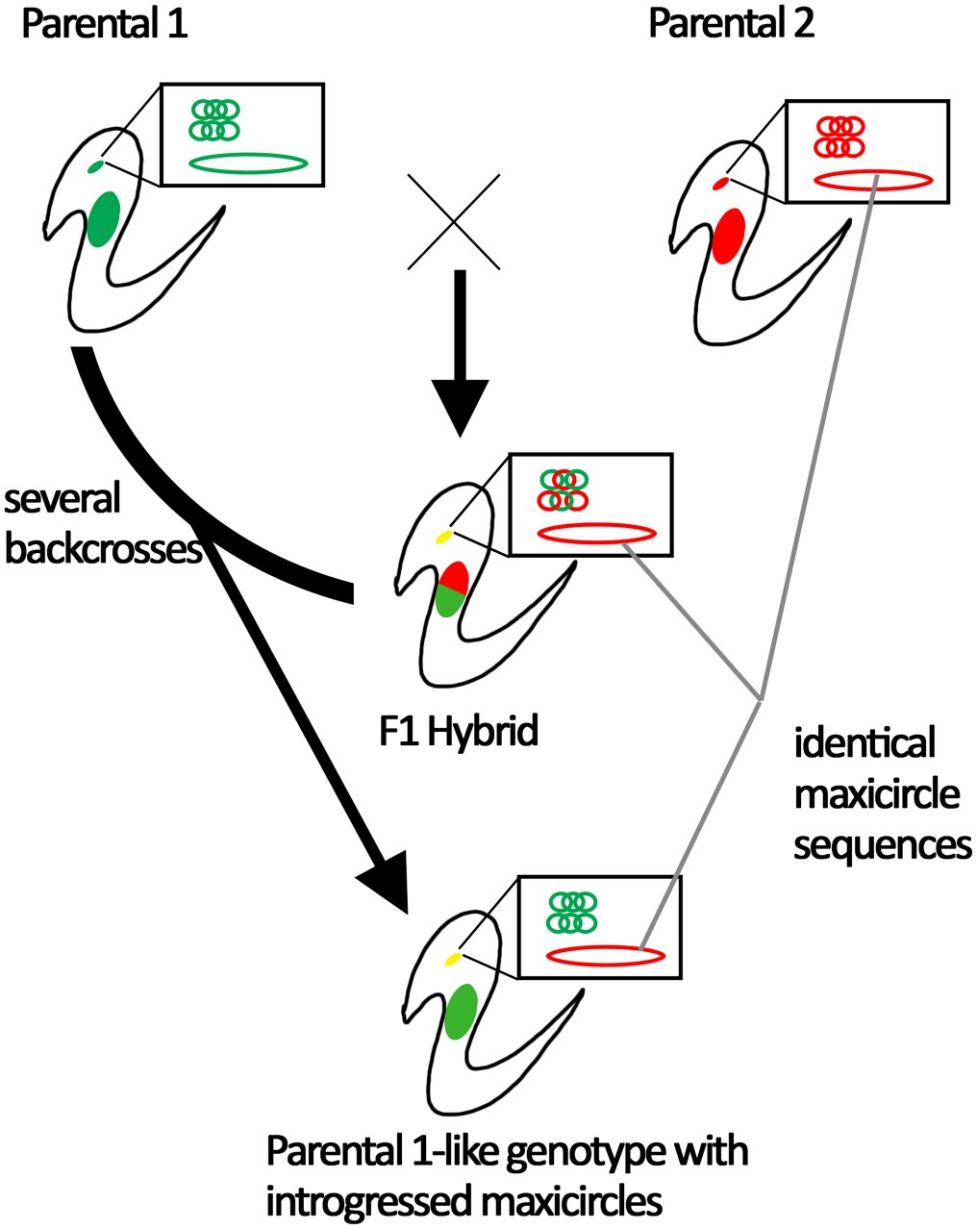
Model explaining shared maxicircles among the TEDa2cl4, TEV55cl1 and PalDa20cl3. Two parental strains with different nuclear genomes (big red and green solid ellipses), different maxicircles (big red and green unfilled ellipses) and different minicircles (catenated small circles) crosses to form an F1 hybrid. The F1 has mixes of minicircles and both nuclear genomes but it has maxicircles from just one parental. Backcrosses of the F1 get the descendants of the F1 more and more similar to the one of the parental genotypes but maintaining the maxicircle from the other parental.

The three strains also gave us the possibility to address kDNA inheritance in hybrids. Maxicircle inheritance is assumed to be uniparental according to previous results in hybrid DTUs TcV and TcVI, since they share maxicircles with TcIII but not with TcII [31, 38]. We were unable to address the hypothesis of uniparental inheritance of maxicircles because the DNA fragments analyzed here were identical among the three strains. In this sense, entire maxicircle sequencing may be required to address ancestry. However, the three strains shared identical maxicircle DNA fragments, and this was surprising since substitution rates are higher in kDNA coding genes than in nuclear ones. Under neutral theory, changes in nuclear and maxicircle DNAs are accumulated with different speed, but the ratio between the magnitude of accumulation is expected to be the same regardless of divergence time between the lineages [37]. This is not the case here. Theoretically, maxicircles could be fixed in the ancestral strain and retained unchanged in the diverged genotypes for stochastic reasons or stabilizing selection. However, this last scenario is unlikely considering the accumulated mutation rate in maxicircles (See Fig 2A). In addition, low divergency in maxicircles was also reported in TcI populations with sexual reproduction [14, 20]. Consequently, mitochondrial introgression, consisting in successive backcrosses of the hybrid with one of the parental genotypes, is the most likely explanation for these observations. The successive backcrosses clean the genome of information from one parental. However, maxicircles from such parental are maintained (see Fig 4). According to this hypothesis, TEV55cl1 and/or PalDa20cl3 are probably descendants of hybrids that backcrossed with one parental, which could explain why such strains share identical maxicircle sequences (Fig 4).

In this work, the inheritance of minicircles was also addressed. We evaluated the number of shared mHVR clusters between strains. We observed that most of the mHVR clusters in TEDa2cl4 (84%) were shared with TEV55cl1 and/or with PalDa20cl3 at 85% identity threshold. In contrast, TEV55cl1 and PalDa20cl3 shared less than 30% of the mHVR clusters. These results support that TEDa2cl4 has minicircles from two different sources corresponding to two different genotypes. Our results also showed that TEDa2cl4 is not a direct descendant of PalD20cl3 and TEV55cl1 considering that there was a 50% of mHVR clusters in TEDa2cl4 that were not present in the studied putative parental strains when a restrictive identity threshold is used (i.e. 99% of sequence identity).

We have previously suggested biparental inheritance of minicircles in TcV and TcVI [33], which, added to the results presented here, suggests that biparental inheritance of minicircles could be the rule in *T*. *cruzi*. Maxicircle and minicircle biparental inheritance has been observed in *T*. *brucei* hybrids. However, maxicircles (less than fifty copies) are homogenized by genetic drift resulting in the loss of maxicircles of one parental in few generations. On the other hand, minicircles have much more copies (thousands) and they resist the fixation effect of genetic drift for more time. Consequently, in *T*. *brucei* maxicircle inheritance is biparental and just seems to be uniparental due to genetic drift [34–36].

Concluding, this paper shows a natural TcI hybrid and it supports that genetic exchange in *T*. *cruzi* likely implies a reductive division such as meiosis. In addition, genetic exchange would also imply kDNA exchange. In this sense, mitochondrial introgression events appear to be frequent in the population, evidencing outcrossing instead of inbreeding. This probably occurs in regions where the different genotypes overlap their distributions. The mechanism of mitochondrial DNA exchange needs to be clarified in future studies in order to determine whether fusion of mitochondria is required to biparental inheritance of minicircles. Further research on meiosis, gametes and experimental F1 hybrids in *T*. *cruzi* is still needed.

## Supporting information

S1 Fig Comparison of averaged uncorrected *p*-distances among nuclear loci and kDNA loci. (TIF)
