# Supplementary figures and images for: Evidence of hybridization, mitochondrial introgression and biparental inheritance of the kDNA minicircles in Trypanosoma cruzi I
Source: PLoS Negl Trop Dis. 2020 Jan 31;14(1):e0007770. doi: 10.1371/journal.pntd.0007770 (PMC7015434; doi:10.1371/journal.pntd.0007770)

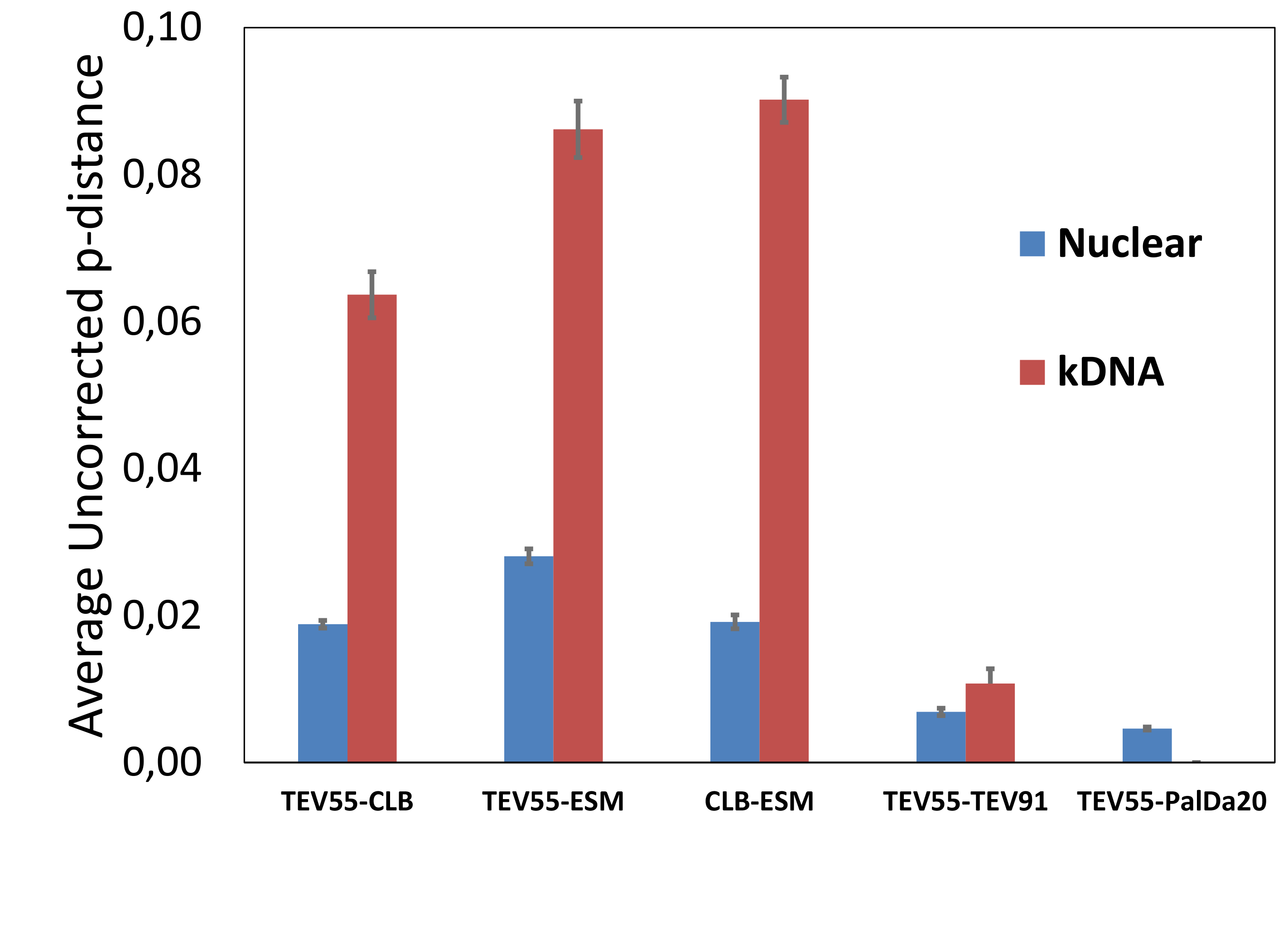

Supplement: S1 Fig — (TIF) [file pntd.0007770.s001.tif]
